# Sonication-assisted activation of empty fruit bunches to produce activated carbon for supercapacitor's electrodes: Surface chemistry and morphology characterization

**DOI:** 10.1016/j.heliyon.2024.e38975

**Published:** 2024-10-04

**Authors:** Egbe Terence Awoh, Joseph Kiplagat, Stephen K. Kimutai, Achisa C. Mecha

**Affiliations:** a Department of Mechanical, Production and Energy Engineering, School of Engineering, Moi University, P.O. Box 3900, Eldoret, 30100, Kenya; b Renewable Energy, Environment, Nanomaterials, and Water Research Group, Department of Chemical and Process Engineering, Moi University, P.O Box 3900, Eldoret, 30100, Kenya; c Department of Electrical and Electronic Engineering, Faculty of Engineering and Technology, University of Buea, P.O Box 63, Buea, Cameroon; d Department of Environmental Science, University of Arizona, Tucson, AZ, 85721, USA

**Keywords:** EFB, Palm biomass, Hydrochar, KOH activation, ultrasonic post-treatment

## Abstract

Thermochemical treatment of empty fruit bunches (EFBs) had been proven to be the fastest way of converting this agro-industrial waste into value. Two-step carbonization and activation was used to convert the EFB to activated carbon with high specific surface area (SSA) and porosity. Hydrothermal carbonization was done at 225 to 275 °C; at retention time of 1–3 h. Activation was done at 800 °C for 1 h, using KOH at two different concentrations. The BET surface area, porosity and surface functional groups of the activated carbon were investigated. The samples experienced change in colourations, with the degree of colouration increases with the time and temperature of the reaction. The increase in these parameters also led to increase in the alkane functional groups and corresponding decreased in the hydroxyl functional groups. The specific surface area was measured using BET and maximum surface of 1375.26 m^2^/g was obtained when the biomass was carbonized at 275 °C for 1 h, and activated at 800 °C using KOH at a concentration of 1:1 to the hydrochar. Ultrasonic post-treatment of the activated carbon enhanced the BET surface area and pore volume to about 1.13 and 1.16 times, respectively. The corresponding pore diameter and volume at this optimum condition were 3.32 nm and 0.73 cm^3^/g, respectively. The material demonstrated a high specific surface area for electronic and ionic diffusions.

## Highlights

• KOH activation of EFB was studied.
• Ultrasonic post-treatment of the AC was used.
• Maximum surface area of 1375 m^2^/g was obtained.

## Graphical abstract

**Figure:**
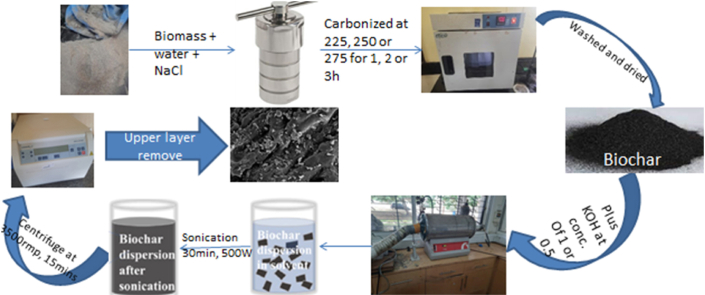

## 1 Introduction

Palm oil is the most affordable and consumed form of vegetable oil in the market today (statista, 2023), on the account that it has the highest yield per hectare; producing about four times rapeseed oil. It is also labour effective and contributing to approximately 39.6 % of the total vegetable oil consume in the world (oilworld, 2019). Presently, palm oil production has also gained interest in the field of biofuel (biodiesel) production [1]. However, palm oil production is limited by two major drawbacks; (i) where it can be grown, and (ii) waste management derived from the processes. Palm oil can only give maximum productivity when grown in the right biophysical conditions, which perfectly describe the equatorial rainforest [2]. At the moment, about 70 % of the rainforests in Indonesia and Malaysia have been cleared for this purposed, which is now driving expansion to Africa. Moreover, palm oil production process leaves behind huge amount of biomass (about 80 % of the overall process) which are not usually being disposed sustainably; especially the empty fruit bunches which account to 23 % of the total production [3,4]. With this agro-industrial scale waste, there is a need to incorporate a feasible and sustainable ways of managing it.

Recent researchers had used conversion methods like pulping, composting, fungiculture, the making of composite, pyrolysis and the production of biofuel. These processes reduces the bulk of the biomass and emission, while producing values [5]. Among all these processes, thermochemical process of pyrolysis/carbonization is the most feasible, the reaction time is short, and the end-product (biochar/biofuel) has wide range of applications [6]. Pyrolysis of palm biomasses have recently caught the attention of many researches nowadays, due to the fact that it provides a feasible means of handling these huge agro-industrial waste. One-step pyrolysis methods have been used to produce biochar (and biofuel) with high calorific values. These biochar obtained from the process could still be used for soil amendment, and could also act as raw material for manufacturing of electrochemical electrodes, depending on the specific surface area (SSA) and porosity of the materials [7].

There are different types of carbonization processes (slow, fast, flash and hydrothermal carbonization) each depending on the operational temperature of the furnace, retention time and heating rates; and the end-products (biochar, bio-oil or biogas) are also dependent of these parameters. However, hydrothermal carbonization (HTC) is a promising way to handle EFBs with high moisture content, and producing high quantity of hydrochar [8]. HTC process also gives the opportunity to incorporate enhancers/catalyzes like sodium salt under high pressure. The effect of sodium salt Na_x_A (Cl^−^, NO^3−^ etc.) on the HTC of biomass had been studied by a number of researchers including the work of Ming et al., [9]. Their results demonstrated that the presence of these sodium salts greatly accelerated the HTC reaction, producing porous carbon-coated nanomaterials. These effects had also been demonstrated by the work of Xu et al., using NaCl as catalyst for the HTC process of rice husks [10]. The carbon material derived from the process had initial cracks on the surface, which was associated to the interaction of the Na^+^ and Cl^−^ with the –OH groups; thereby improving the carbonization process due to the enhanced hydrolysis of cellulose and hemicellulose.

Biochar can further undergo a process known as activation, to increase its specific surface area (SSA) and porosity, given rise to activated carbon (AC) [11]. Usually, activation is done in the presence of steam/carbon dioxide (physical activation), or with chemical activating agents (chemical activation) like KOH, H_3_PO_4_ and others. Chemical agents like KOH are typically used in large scale production due to their affordability, and the ability to produce activated carbon with high and well-developed micro and mesoporous structures as compared to other activating agents like ZnCl_2_ or H_3_PO_4_ [12]. KOH activated carbons are also known to exhibit ultrahigh specific surface area of up to 3000 m^2^/g [13,14]. KOH have this edge over other chemical agents because of the ease on which K can intercalate with the carbon atoms, forming rigid porous structures that are capable of maintaining its form even after removal of the intercalated K [13].

Different types of palm biomasses have been treated using this activating agent [15]. Two-step carbonization of EFB using KOH activating agent have been done by some researchers. Baktiar et al., used the pyrolysis/activation utilizing KOH to produce activated carbon. Maximum surface area of 920 m^2^/g was obtained at optimum temperature of 800 °C [16]. This could be associated to the reaction of KOH which starts at a temperature of 700 °C. Therefore, at lower temperature of 400 and 600 °C there is possibility of lower activation. Moreover, one-step KOH activation (800 °C) could also be used to produce activated carbon from EFB; Kurniawan et al., used this method to produce activated carbon with high specific area of about 1309 m^2^/g [17]. This is mainly because KOH is a strong base that supports the single step process. Even though one-step activation seems easier; however, the activation time and KOH concentration is usually higher, making it more expensive.

These physiochemical properties (specific surface area and pore volume) that make activated carbon great material for manufacturing of supercapacitor's electrode can be further enhanced by sonication (ultrasonic treatment) [18]. Sonication is a mechanical process that encourage the generation and collapsing of high pressure bubbles to create cavitation in the activated carbon. This thus grows the pores size and increases the specific surface area. This results have been supported by many researchers like Liu et al., who recorded a 1.18 increased in the specific surface area of pristine activated carbon after sonicated at 40 kHz for 5 min [19]. Similarly, Fröhlich et al., used ultrasonic post-treatment of activated carbon at 24 kHz, 400 W for 1 h. The sonicated AC experienced increased in total pore volume and SSA to approximately 1.36 and 1.144 times, respectively [18]. Chin et al., also used sonication post-treatment to enhance the surface morphology and SSA of coconut shell activated carbon, due to the cavitation clearing the pores [20]. The effect of sonication temperature was study as well, and it was discovered that temperature of 40 °C optimal for the reaction. Liu et al., used this technique to enhance the SSA and pore volume of carbon; they reported that the mechanical vibration and cavitation encourage the conversion of micropore to mesopore [21]. Similarly technique of sonication post-treatment is also used in activated carbon regeneration. Cao et al., demonstrated this phenomenon in their work, resulting to increase of SSA and pore volume to up to 8.6 and 8.8 times respectively, as compare to the untreated AC. This was attributed to the cleaning of the surface and enlargement of the pores by removal of impurities and ash on the pristine activated carbon [22,23].

The novelty of this research lies in providing an affordable technique to produce activated carbon with high specific surface area and porosity from Empty fruit bunches (EFBs). Sodium chloride (NaCl), an affordable catalyst was used during the hydrothermal carbonization to produce high quantity and quality hydrochar. Lower concentrations of potassium hydroxide were used at the same temperature of 800 °C to produce the highly porous activated carbon with high specific surface area. Sonication post-treatment of the pristine activated carbon was done for 30 min at a temperature of 40 °C, and centrifugation at 3500 rpm for 30 min.

## 2 Materials and methods

### 2.1 Preparation

**Fig. 1:**
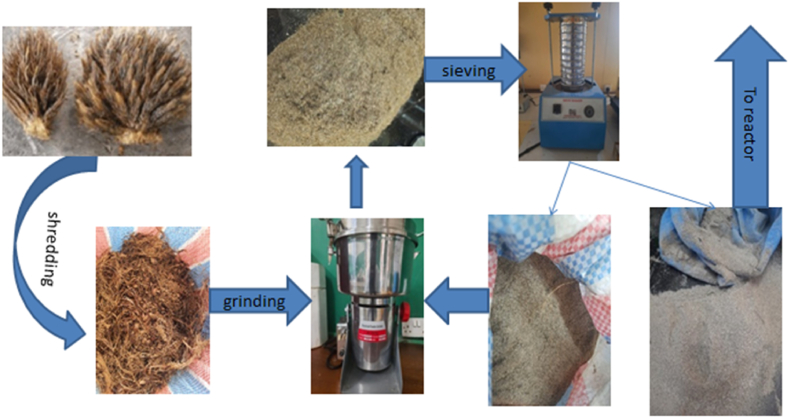
Empty palm biomass processing.

The empty fruit bunches used in the experiments were taken from Idenau (Cameroon) oil mill, hand-shredded to reduce the size, and then washed with distilled water to remove dust and impurities. The raw material was dried in an oven at 80 °C for 15 h to complete dryness. The biomass was then milled with a biomass powder grinder (DE-2000g), before sieving with laboratory sieve shaker (Liya LT-G0023, Kenya) of aperture 100 μm to have the raw materials used in the carbonization process. Fig. 1 below summaries the biomass processing step.

### 2.2 Carbonization process

The hydrothermal carbonization process was done using a 250 ml autoclave hydrothermal reactor. The processed biomass, sodium chloride (NaCl) and distilled water were mixed at a ratio of 1:1:3, with each batch containing 50 g of biomass. The reactor was carbonized using box furnace (Carbolite Gero 30–3000, UK) at three different temperatures (225, 250 and 275 °C). The heating rate was set at 5 °C/min. Three different holding times (1, 2 and 3 h) were observed in the experiments. After the said time and temperature, the reactor was let to cool natural to room temperature. The hydrochar was removed from the reactor after each process, washed with distilled water and dried at a temperature of 80 °C to complete dryness. The percentages yielded by the carbonized samples were calculated using Equation (1). The carbonized biomass was code with letter ‘A’ to ‘I’ depending on the carbonization temperature and reaction time used as shown in Table 1. The experiments were done in multiple to ensure repeatability.

(1) $$\%yield=\frac{Mass\;after\;carbonization}{Mass\;before\;carbonization}\times 100$$

**Table 1:** Coding of biochar after carbonization.

	225 °C	250 °C	275 °C
1 h	A	D	G
2 h	B	E	H
3 h	C	F	I

### 2.3 Activation process

The activation of the samples were done using potassium hydroxide (KOH, Merck), at two different concentrations. The KOH was completely dissolved in 50 ml of distilled water under stirring before adding 6 g of the sample, and continuous stirring to mix well. The mixture was then placed in an oven at temperature of 80 °C for about 15 h, to dry completely. The mixture was transfer to a boat crucible before inserting into a tube furnace (Carbolite Gero TZF/12/38/400, UK) for the activation process. Activation process was done at 800 °C, ramping at a heating rate of 5 °C/min. All activations were done in the presence of Nitrogen flowing at a rate of 60 cm^3^/min. The reaction time was set to 1 h, after which the reactor was turn off to cool naturally. The sample was then treated with 0.1M HCl acid, and washed with distilled water until the pH was neutral; then dried and weighted. The percentages yielded by the activated samples were calculated using Equation (2). The samples were label according to the letter given after HTC, and according to the concentration of KOH used in the activation.

(2) $$\%yield=\frac{Mass\;after\;activation}{Mass\;before\;activation}\times 100$$

### 2.4 Sonication and centrifugation process

**Fig. 2:**
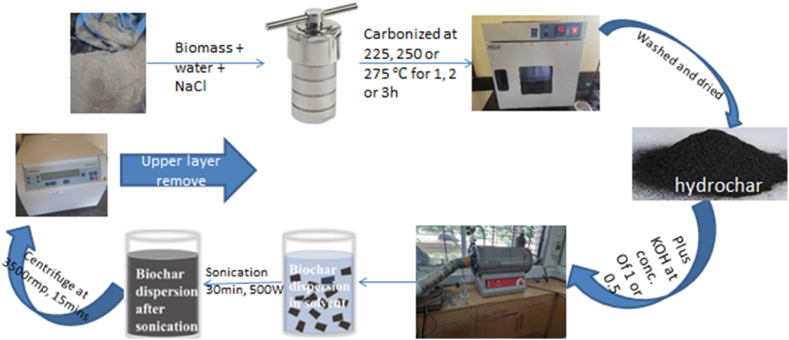
Biomass conversions into porous activated carbon process.

2 g of the activated sample was placed in a beaker containing 250 ml of distilled water. The sample was sonicated at 40 kHz (500 W, 40 °C) for 30 min using bath sonicator (ULTR-30L-001). The mixture was then centrifuged (VWR Mega Star 1.6/1.6R) at 3500 rpm for 15 min. The upper layer of the solution was siphoned out and dried at a temperature of 70 °C, to complete dryness. The samples were packed in an air-tight container for testing. Fig. 2 below summaries the conversion processes.

### 2.5 Characterization

The Fourier transform infrared (FTIR) spectrum of the carbonized samples and raw empty fruit bunch powder were carried out using the FTIR (IRSpirit QATR-S) spectrometer at a range of 4000 to 400 cm^−1^.

The morphology of the activated carbon was determined by the physical adsorption of nitrogen at 77 K using NOVA 1200e surface area and pore size analyzer (Quantachrome). About 0.2 g of each sample was degassed using nitrogen at 200 °C for 4 h and the BET method was used to directly measure the specific surface area (S_BET_) at relative pressure ranging from 0.06 to 0.18. The pore size distribution and volume were determined using the ISO 9277:2010 and IUPAC standard (IUPAC, 2015). The Zeiss high resolution electron microscope (Zeiss Sigma 360V) was used to examine the morphological properties of the activated carbon capture at a voltage of 5.0 kV.

## 3 Results and discussion

### 3.1 Effect of carbonization parameters

#### 3.1.1 Temperature

**Fig. 3:**
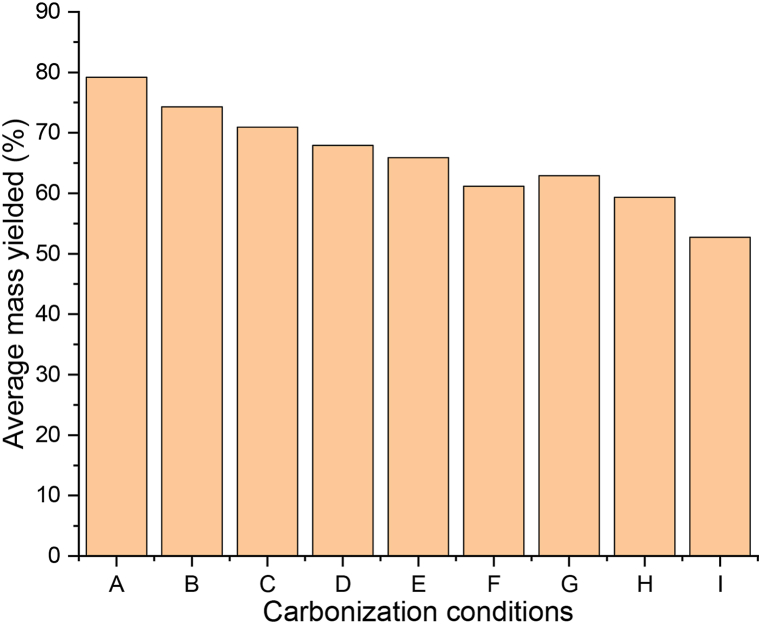
Percentage of hydrochar yielded after carbonization.

**Fig. 4:**
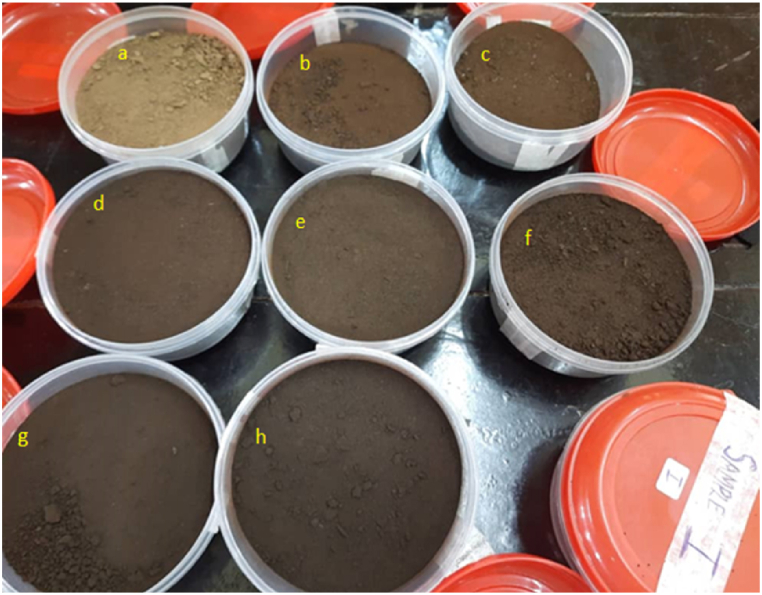
Image of the samples after carbonization.

**Fig. 5:**
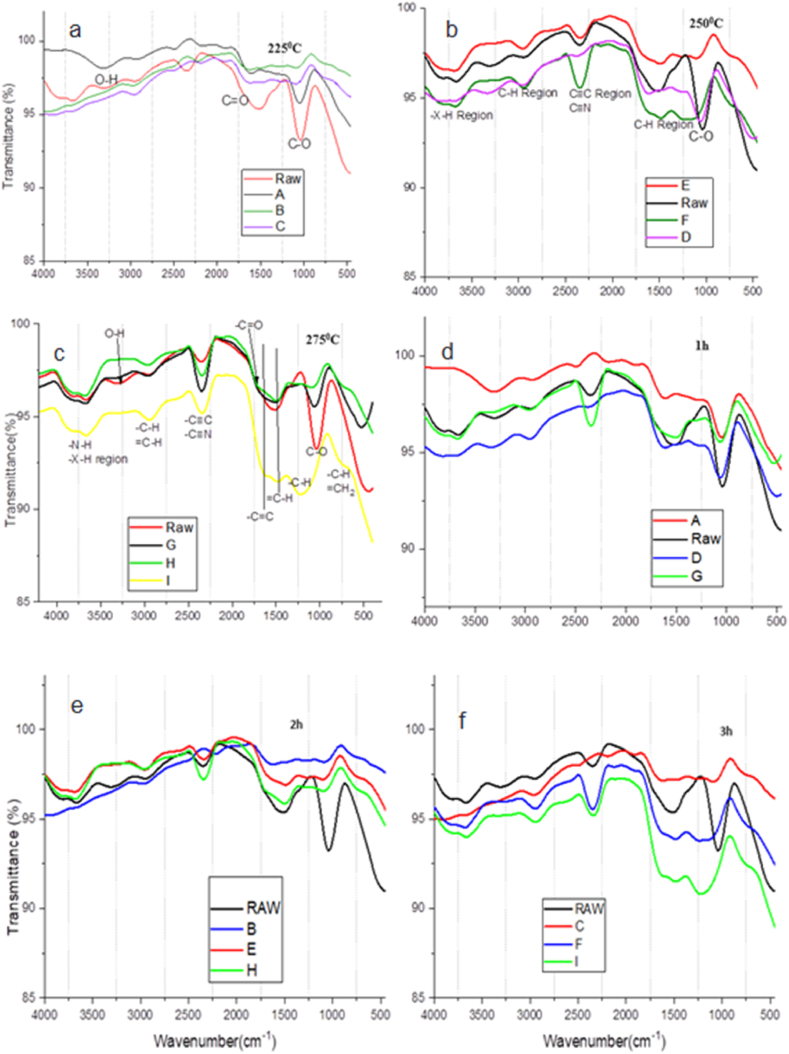
FTIR spectral of the carbonized EFB samples at the; a) same temperature of 225 °C; b) same temperature of 250 °C; c) same temperature of 275 °C; d) same reaction time of 1h; e) same reaction time of 2h; f) same reaction time of 3h.

The biomass underwent continuous degradation with temperature as shown in Fig. 4. The samples tend from dark brown to a darker colouration when view with respect to temperature, indicating that as the temperature increases, so too was the degree of carbonization [24,25]. This is due to the increase in the carbon content or alkane functional group, and the corresponding reduction in the oxygen functional group as supported by the FTIR spectral (Fig. 5a–c). Fig. 3 shows the mass of hydrochar yielded after carbonization. Percentage yield was obtained using Equation (1), and gives the conversion efficiency of 52.74–79.2 % which is line with the recent literatures on hydrothermal conversion processes [26].

There is also a progressive decreased in mass as the hydrothermal temperature increases from 225 to 275 °C. This is because of the greater loss of mass as volatile at higher temperatures due to the increase in the secondary polymerizations [27]. The carbon content also increases with increase in hydrothermal temperature, as supported in Fig. 4, Fig. 5 and 5 (a-c). Sample ‘I’ has the less conversion mass of 52.74 %. The loss in mass was due to the release of volatile matter from the decomposition of the hydroxyl group, and was directly proportional to temperature [28,29].

#### 3.1.2 Retention time

A progressive change in colouration could also be seen when the samples are observed with respective to retention time (Fig. 4); indicating the degree of carbon content, or increased in carbonization with retention time. There is also a progressive decreased in mass as the hydrothermal retention time increases from 1 to 3 h (Fig. 3). Longer retention time often yielded greater carbon content due to the severity of the reaction with time, but there is a corresponding loss of mass as volatile (Fig. 5c–f) [30]. In general, longer retention act like high temperature, but the effect of temperature is greater as compare to the retention time [31].

### 3.2 FTIR of carbonized sample

The spectral of the raw EFB biomass and the carbonized samples shows many peaks characterized by the different functional groups (Fig. 5a–f). Characteristic peak of the raw EFB shows –OH (hydroxyl group) stretching vibration at around 3300 cm^−1^, –C-H bonds common in cellulose and hemicellulose at a peak of 2800 cm^−1^ [32]. The shoulder band that appear at 1716 cm^−1^ is based on the carbonyl group (–C=O) from the stretching of fatty acids in the EFB fiber. Aromatic carbons, –C-O are associated with the stretching vibration at 1048 cm^−1^ [33].

As the carbonization temperature and reaction time increases, the intensity of –C-O stretching vibration at 1048 cm^−1^ is disappearing. On the other hand the intensity of the –C=C- stretching vibration, =C-H bending vibration, and -C-H bending vibration at 1615, 1505 and 1216 cm^−1^ respectively are increasing [34,35]. The formation of new peak at 714 cm^−1^ associating to –C-H bending vibration and = CH_2_ wagging vibration can also be observed, and the intensity of these peaks also increases with temperature and reaction time [36]. Similarly, the C-H and C ≡ C stretching vibration region peaking at 2921 cm^−1^ and 2332 cm^−1^ respectively, experiences growth in intensity as the temperature and retention time increases [37]. The oxygen containing group including hydroxyl group give off the oxygen and are release in the form of the oxide during heating [38]. The free H and C radicals combined to form methyl group, and other –C=C- and C ≡ C functional groups [39].

### 3.3 BET analysis

The mass of the resultant activated carbon obtained varied with respect to the initial carbonization conditions; with sample I having the least lost unlike in the case of the carbonization (Table 3). This is due to the fact that lesser mass is lost during graphitization as volatile, depending on how much was already lost during carbonization [40,41]. The activation yield was in the range of 46.33–65.33 %, calculated using Equation (2).

All samples attained full activation with the BET specific surface area of the samples after sonication in the range of 1268–1375 m^2^/g (Table 3). This was greater than the commercial activated carbons which are usually in the range of 500–1000 m^2^/g (Table 2). Maximum specific surface area of 1375.26 m^2^/g was obtained when the EFB was carbonized at 275 °C for 1 h, before activated with KOH at a ratio of 1:1 to the hydrochar.

**Table 2:** Recent researchers on the use of Biomass to produce activated carbon.

Palm Biomass	1st Reactor condition	2nd Reactor condition	S_BET_ (m^2^/g)	Ref
EFB		Steam, 765 °C, 77min	720	[43]
EFB	N_2_, 400 °C, 60min	CO_2_ & N_2_, 900 °C, 60min	869	[44]
EFB	N_2_, 450 °C, 90min	H_3_PO_4_, 600W, 10min	700	[45]
EFB	KOH, 350 °C, 120min	KOH, 800 °C, 45min	920	[16]
EFB		KOH, 800 °C, 180min	1309	[17]
Palm kernel shell	KOH, 800 °C, 120min		1160	[46]
Palm kernel shell	KOH, 800 °C, 120min	KOH, 800 °C, 120min	1298	[46]
Kanlow switchgrass	500 °C, 30min	KOH, 900 °C, 60min	1272	[7]
Miscanthus	500 °C, 30min	KOH, 900 °C, 60min	1597	[7]
Rice straw	KOH,800 °C, 60min		866	[47]
Rice straw	800 °C, 60min	KOH,800 °C, 60min	1444	[47]
Giant reed	KOH, 600 °C, 120min		1122	[48]
Rice husk	600 °C, 120min	KOH, 600 °C, 60min	755	[49]
Commercial AC			500–1000	[[50], [51], [52]].
EFB	NaCl, 275 °C, 60min	KOH, 800 °C, 60min	1375	This work

The porosity of the carbon is greatly affected by the activation mechanism of both NaCl and KOH. The addition of NaCl during hydrothermal carbonization promotes the elimination of hemicellulose and enhanced depolymerization of lignin. These helps in the formation of monomers during the HTC and hence contributed in the formation of initial cracks on the hydrochar's surface [9,10]. The cracks form during the carbonization process is grown during the chemical activation with KOH. At high temperature, KOH decomposed and releases gaseous derivatives according to equations [[3], [4], [5], [6], [7], [8]] below, which then act on the already existing pores [42]. The pores are then forced-opened when these gaseous derivatives are escaping, and therefore increased the surface area of the char.Table 3

(3) $$KOH\;\Delta -\to {\;K}_{2}O+H_{2}$$

(4) $$Palm\;biomass_{\left(s\right)}\left(C_{x}H_{y}O_{z}\right)+{\;K}_{2}O\;\Delta -\to {\;C}_{x}H_{y-2}O_{z}+char\left(C\right)+H_{2\left(g\right)}+{tar}_{\left(s\right)}$$

(5) $$char\left(C\right)+H_{2}O_{\left(steam\right)}\;\Delta -\to H_{2\left(g\right)}+C$$

(6) $$CO+H_{2}O_{\left(steam\right)}\;\Delta -\to H_{2\left(g\right)}+CO_{2\left(g\right)\;}$$

(7) $${\;K}_{2}O+char\left(C\right)\Delta -\to 2K+CO_{\left(g\right)\;}$$

(8) $${\;K}_{2}O+CO_{2\left(g\right)}\;\Delta -\to {\;K}_{2}C$$

**Table 3:** Morphology and surface chemistry of the activated carbon.

code	Activation yield(g)	BET(m^2^/g)	Average Pore diameter (nm)	Total Pore vol (cm^3^/g)	Mesopore vol (cm^3^/g)
A1	2.78	1298.36	3.72	0.67	0.53
C0.5	2.91	1286.55	2.98	0.52	0.42
D0.5	3.27	1268.76	2.83	0.47	0.41
D1	3.21	1289.56	2.95	0.61	0.57
E0.5	3.53	1278.91	2.65	0.48	0.39
F1	3.5	1309.28	3.60	0.68	0.59
G1	3.62	1375.26	3.32	0.73	0.59
H0.5	3.78	1280.29	4.44	0.50	0.43
H1	3.89	1370.63	3.11	0.71	0.65
I0.5	3.92	1288.52	3.13	0.56	0.49
Unsonicated G1		1218.07	3.17	0.63	0.42

The mechanical stress of sonication induces cavitation, which also improve the surface properties of the AC via the introduction, growth and vigorous collapsing of high pressure bubbles in the AC. This also led to increase in the specific surface area and porosity of the activated carbon [18,21]. Before the vigorous blasting of sample G1 by ultrasonic wave, the specific surface area and total pore volume of the sample were 1218.07 m^2^/g and 0.64 cm^3^/g, respectively. Thus, the sonication post-treatment increased the specific surface area and pore volume of the activated carbon by approximately 1.13 and 1.16 times, respectively.

**Fig. 6:**
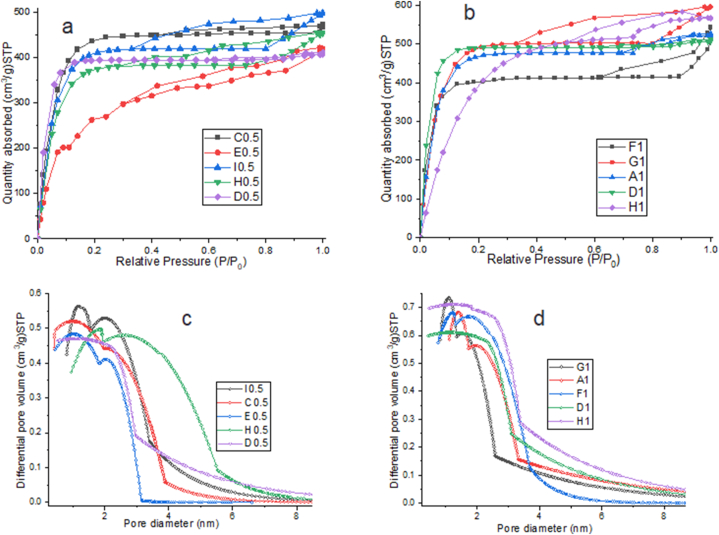
N_2_ adsorption isotherms of sample activated with a) ratio of 1:0.5; b) ratio of 1:1; and pore size distribution sample activated with c) ratio of 1:0.5; d) ratio of 1:1.

The Nitrogen adsorption-desorption isotherms of all EFB porous activated carbon are presented in Fig. 6(a–b). The samples showed high nitrogen adsorption at low relative pressure, a behave common with type I isotherms, indicating the presence of microporous structure (1–2 nm) in the samples [53]. All samples also exhibited certain degree of hysteresis in their isotherms, characterized by the presence of mesoporous structure (2–50 nm) in the materials, a behavior common with type IV isotherms [54]. Therefore, all isotherms were combination of type I and IV, hence the activated carbon were characterized by microporous and mesoporous structure [55]. The pore size distribution of the samples (Fig. 6c–d) showed bimodal distribution with two peaks closed to 1 nm and 2 nm. The average pore diameters of the samples were in the range of 2.65–4.44 nm (Table 3).

### 3.4 SEM and EDS analysis

**Fig. 7:**
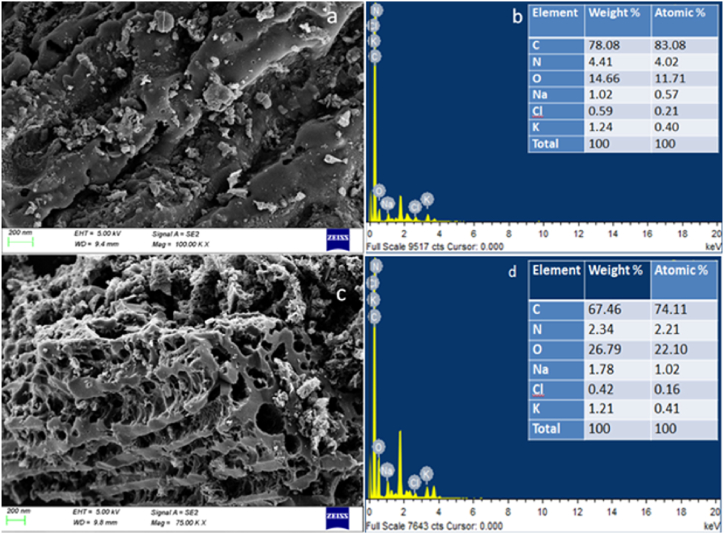
Structural morphology of the samples; (a) SEM data for G1; (b) EDS data G1; (c) SEM data for H1; (d) EDS data for H1.

Fig. 7 showed the EDS and SEM characteristic of sample G1 and H1. The EDS spectral of G1 and H1 showed high percentages of carbon (78.08 % wt for G1 and 67.46 % wt for H1). The samples also showed high oxygen content in their structure, at a ratio of 14.66 and 26.79 % wt for G1 and H1, respectively. The C/O ratio of both samples were 5.33 and 2.52, respectively for sample G1 and H1 [56].

Traces of Sodium (Na), Chlorine (Cl) and Potassium (K) are also present, which could have been originated from the usage of inorganic fertilizers at some stage of the plant growth [57]. Activating with KOH create gaseous derivative that escape and forced open the pores that where already present during carbonization, and thereby increasing their density.

## 4 Conclusion

Sonication-assisted activation of empty fruit bunches was undertaken to produce activated carbon. The materials were characterized using FTIR, BET, SEM and EDS spectroscopy. Based on the FTIR analysis, temperature had a greater effect on the EFB hydrochar production as compared to retention time. Both temperature and retention time influenced the functional groups; and the degree of intensity of the methyl group, –C=C- and -C ≡ C- formation increases with these parameters. Similarly, the mass of the hydrochar yielded decreased with increasing temperature and retention time due to loss of volatile matter. The morphology of the samples showed high carbon to oxygen ratio similar to that of reduced graphene oxide. All activated samples showed high specific surface area in the range of 1268≤S_bet_≤1375 m^2^/g and high porosity which was comparative to other activated carbon produced using different method, activating agents and biomass. Ultrasonic post-treatment also assisted in enhancing the physiochemical properties of the activated carbon. The high S_BET_ could offers a less resistance path for ionic and electronic diffusions makes the material suitable for the construction of supercapacitor's electrode.

## Data availability statement

Data will be made available on request.

## CRediT authorship contribution statement

**Egbe Terence Awoh:** Writing – original draft, Software, Methodology, Investigation, Formal analysis. **Joseph Kiplagat:** Writing – review & editing, Validation, Supervision, Funding acquisition, Formal analysis, Data curation, Conceptualization. **Stephen K. Kimutai:** Writing – review & editing, Visualization, Supervision, Methodology, Data curation. **Achisa C. Mecha:** Writing – review & editing, Visualization, Validation, Supervision, Resources, Project administration, Methodology, Investigation, Funding acquisition, Data curation, Conceptualization.

## Declaration of competing interest

The authors declare the following financial interests/personal relationships which may be considered as potential competing interests: Egbe Terence Awoh reports financial support, statistical analysis, travel, and writing assistance were provided by MIRET. If there are other authors, they declare that they have no known competing financial interests or personal relationships that could have appeared to influence the work reported in this paper.
